# Photonic Biosensor Assays to Detect and Distinguish Subspecies of *Francisella tularensis*

**DOI:** 10.3390/s110303004

**Published:** 2011-03-07

**Authors:** Kristie L. Cooper, Aloka B. Bandara, Yunmiao Wang, Anbo Wang, Thomas J. Inzana

**Affiliations:** 1 Center for Photonics Technology, Virginia Polytechnic Institute and State University, Blacksburg, VA 24061, USA; E-Mails: klcooper@vt.edu (K.L.C.); kathy618@vt.edu (Y.W.); awang@vt.edu (A.W.); 2 Center for Molecular Medicine & Infectious Diseases, Virginia Polytechnic Institute and State University, Blacksburg, VA 24061, USA; E-Mail: abandara@vt.edu (A.B.B.)

**Keywords:** *Francisella tularensis*, diagnosis, long period fiber grating, fiber Fabry-Perot interferometric sensor, probe, antibody, DNA

## Abstract

The application of photonic biosensor assays to diagnose the category-A select agent *Francisella tularensis* was investigated. Both interferometric and long period fiber grating sensing structures were successfully demonstrated; both these sensors are capable of detecting the optical changes induced by either immunological binding or DNA hybridization. Detection was made possible by the attachment of DNA probes or immunoglobulins (IgG) directly to the fiber surface via layer-by-layer electrostatic self-assembly. An optical fiber biosensor was tested using a standard transmission mode long period fiber grating of length 15 mm and period 260 μm, and coated with the IgG fraction of antiserum to *F. tularensis*. The IgG was deposited onto the optical fiber surface in a nanostructured film, and the resulting refractive index change was measured using spectroscopic ellipsometry. The presence of *F. tularensis* was detected from the decrease of peak wavelength caused by binding of specific antigen. Detection and differentiation of *F. tularensis* subspecies *tularensis* (type A strain TI0902) and subspecies *holarctica* (type B strain LVS) was further accomplished using a single-mode multi-cavity fiber Fabry-Perot interferometric sensor. These sensors were prepared by depositing seven polymer bilayers onto the fiber tip followed by attaching one of two DNA probes: (a) a 101-bp probe from the *yhhW* gene unique to type-A strains, or (b) a 117-bp probe of the *lpnA* gene, common to both type-A and type-B strains. The *yhhW* probe was reactive with the type-A, but not the type-B strain. Probe *lpnA* was reactive with both type-A and type-B strains. Nanogram quantities of the target DNA could be detected, highlighting the sensitivity of this method for DNA detection without the use of PCR. The DNA probe reacted with 100% homologous target DNA, but did not react with sequences containing 2-bp mismatches, indicating the high specificity of the assay. These assays will fill an important void that exists for rapid, culture-free, and field-compatible diagnosis of *F. tularensis*.

## 1. Introduction

*Francisella tularensis* is a tiny, pleomorphic, gram-negative coccobacillus responsible for epizootics of tularemia. *F. tularensis* subspecies *tularensis* (type A) is the primary etiologic agent, and the most virulent subspecies. Other subspecies include *holarctica* (also highly virulent), *novicida*, and *mediasiatica* [1]. The disease cycle of *F. tularensis* is maintained in nature between wild animals, biting vectors, and the contaminated environment (primarily aqueous). Transmission to humans occurs through handling of or ingesting infected animals or water. Ticks, some biting flies, and other arthropods are important vectors that may also transmit tularemia to animals and humans [2]. In the United States between 1990–2000, 86–193 cases of tularemia occurred per year for a total of 1,368 cases from 44 states [3]. *F. tularensis* is highly virulent (as few as 10–50 organisms can cause an infection in humans), and can survive for long periods under harsh environmental conditions [2]. *F. tularensis* has been identified by the Centers for Disease Control and Prevention (CDC) as a Category-A select agent (CDC Strategic Planning Workshop, 2000) because it is easily transmitted, can inflict substantial morbidity and mortality on large numbers of people, and can induce widespread panic [4,5].

A delay in diagnosis of tularemia and late administration of effective antibiotic therapy results in increased morbidity and mortality. Without treatment, nonspecific symptoms usually persist for several weeks [6]. However, culture requires the availability of BSL-3 facilities, and even with such facilities, identification can be difficult (particularly by laboratories unfamiliar with the agent) and is very time consuming. As a result, tularemia has often been diagnosed by serological tests, such as tube agglutination, adapted microagglutination [7,8], and enzyme-linked immunosorbent assay (ELISA) [9,10]. However, serological assays normally require at least two weeks after infection to support a diagnosis. Fluorescent staining and antigen detection by antibodies with enzymatic tags is available, but these tests require sophisticated equipment and are not considered rapid (immuno-histochemistry, fluorescence microscopy, and Western blotting). Due to the potential for intentional release of *F. tularensis* as a bioweapon, an assay to rapidly and accurately identify this agent for the military deployed in undeveloped countries, or for civilians, is needed.

Pohanka and Skladal [11] developed an immunosensing device based on a piezoelectric sensor for direct detection of *F. tularensis*. This sensor included mouse polyclonal antibody immobilized in a layer of protein A covalently linked to the gold electrode of the sensor. The immunosensor was able to detect a limit of 10^5^ colony forming units (CFU)/mL of *F. tularensis*. The sensor was successfully evaluated for rapid detection of *F. tularensis* spikes in drinking water and milk. Chinowsky *et al*. [12] developed compact multi-analyte surface plasmon resonance (SPR) instruments based on Texas Instruments' Spreeta sensing chips to detect biological warfare agents including *F tularensis*. These instruments allowed a sample to be screened for up to 24 different substances simultaneously, and an antigen-antibody based SPR instrument was able to successfully detect *F. tularensis* at greater than 10^4^ CFU/mL. Taitt *et al*. [13] developed a fluorescence-based multianalyte immunosensor for simultaneous analysis of multiple samples. This antigen-antibody assay was successful in detecting nine infectious agents, including *F. tularensis* LVS. O’Brien *et al*. [14] developed, optimized, and evaluated a bidiffractive grating biosensor (BDG) as a potential field deployable biosensor. Well-characterized immunochemical reagents were employed in developing the assays in the BDG for detection of four separate agents, including *F. tularensis*. Thus far most sensor-based assays have used an immunological approach to detect bacterial antigens. They have not been developed for detecting bacterial DNA in samples without PCR amplifications.

The Center for Photonics Technology at Virginia Tech has developed optical sensors for a wide range of analytes such as temperature [15], pressure [16,17], flow [18], acoustics [17,19], humidity, and various gases [20,21], including the world’s smallest high temperature pressure sensor [22]. The layer-by-layer electrostatic self-assembly (LbL/ESA) process allows direct attachment of these probes to the fiber surface, either the endface or side surfaces. The result is that either immunoglobulins (IgG) or DNA may be used for both interferometric and long period fiber grating sensing schemes. In this communication, we report the development of biosensor prototypes that incorporate label-free, specific antibodies and single-stranded oligonucleotides for detection of *F. tularensis*, and differentiation of subspecies types A and B. The assay was highly sensitive and specific, and has promise as a diagnostic test to rapidly detect *F. tularensis* under field or simple laboratory settings.

## 2. Experimental Section

### 2.1. Layer-by-Layer Electrostatic Self-Assembly Processing

The layer-by-layer electrostatic self-assembly (LbL/ESA) process was adapted to incorporate antibodies and oligonucleotide probes specific to *F. tularensis* [23–26]. Careful organization of biomolecules into thin-film structures can be achieved by molecular self-assembly using layer-by-layer adsorption of polyelectrolytes. LbL/ESA films are composed of two (or more) polyions, salts, and a substantial amount of water [27,28]. Because the polymer chain is flexible, it is free to orient its geometry with respect to the substrate, so a relatively low-energy, stable configuration is achieved. Film characteristics are dependent on the composition of each monolayer, the process parameters used to deposit the monolayers, and the order in which the layers are assembled [27,29,30]. Previous work focused on the selection of process parameters to minimize thickness variation and maximize polymer refractive index (RI) within the possible range in order to achieve a composite RI in the high-sensitivity LPFG range of 1.400 to 1.452 [29,31]. The same process was used for both substrate types (planar single-crystal silicon and quartz test substrates, and optical fiber sensors), modified only to accommodate the fiber size and geometry, which merely required smaller solution volumes.

Substrates were prepared for thin film deposition in a piranha bath (H_2_SO_4_:H_2_O_2_, 7:3 v/v) for 1 h and rinsed three times with ultrapure water, producing a negatively charged surface. Water used in all experiments had a resistance greater than 18 MΩ cm (Barnstead Diamond RO, Nanopure Diamond UV/UF). To improve self-assembly growth characteristics and ameliorate denaturation [32], a polyelectrolyte film was assembled on each substrate prior to DNA/IgG assembly.

Polyelectrolytes were obtained from Aldrich and used in aqueous solutions of 100–150 g/mL, 0.02 M NaCl, and pH in the range 5.5–7.4. Briefly, a freshly rinsed sensor was immersed in an aqueous poly allylamine hydrochloride (PAH) or poly diallyldimethyl ammonium chloride (PDDA) solution for 5 min, followed by rinsing three times in ultrapure water. It was then immersed in a similar aqueous solution of poly-sodium-4-styrenesulfonate (PSS) for 5 min, and rinsed again. These two steps were repeated alternately with 1 min immersion times until the desired number of bilayers was achieved, typically 6–12, and ending with a cationic layer. The substrate was then dried in a stream of nitrogen and stored at room temperature. The IgG monolayer or single-stranded DNA probes (50 μg/mL) were deposited in a similar manner over a period of 1 h. The experiments described here were performed without the additional step of immersion in a binding blocker. Should nonspecific binding become a concern, the fiber sensor can be immersed in a standard binding block solution, e.g., bovine serum albumin in phosphate buffered saline, pH 7.4 with NaN_3_ as a preservative (PBS-1% BSA-0.1% NaN_3_), as a final sensor fabrication step.

### 2.2. Production and Isolation of Antibodies

New Zealand White Rabbits were inoculated subcutaneously with 10^9^ CFU of irradiated cells of *F. tularensis* live vaccine strain (LVS) until a high titer (ELISA titer > 1:10,000) was obtained, as described elsewhere [33,34]. The IgG component of the immune serum was isolated by Protein-A affinity chromatography, immediately dialyzed against PBS and concentrated by ultrafiltration to approximately 10 mg/mL with a Minicon ultrafiltration device (Amicon Corp., Danvers, MA) [35]. Previous experiments indicated that the majority of this antibody was directed to lipopolysaccharide (LPS) [36].

### 2.3. Long Period Fiber Grating (LPFG) Sensor Operation

Long-period fiber gratings (LPFG) represent a promising technology for numerous optical sensing applications due to characteristics such as compactness, environmental-sensitivity and high resolution, and have been demonstrated for temperature, strain, bending and immuno-sensors. Their operating principle is based on the coupling of light between co-propagating modes of an optical fiber caused by a photo-induced refractive index grating in the optical fiber core. In a single LPFG, a fundamental core mode (LP_01_) is coupled to co-propagating cladding modes (LP_0i_) with the same symmetry. The coupled cladding mode is usually absorbed or scattered out by a fiber polymer coating layer outside the cladding region, resulting in resonant loss peaks in the transmission spectrum. The location of those peaks is dependent on the grating characteristics and the environment immediately surrounding the cladding, including temperature, pressure and refractive index. The refractive index sensitivity makes them strong candidates for optical biosensing applications, based on the synthesis of a biological receptor nanostructure on the cladding surface. Antigen binding produces an increase in refractive index, shifting the resonance peak to lower wavelengths. In general, as the external index increases from 1.00 to the cladding index, the resonant wavelength is blue shifted, most dramatically as the index approaches the cladding index. Sensitivities of 0.0001 refractive index units (RIU) are possible, which corresponds to fM quantities of analyte. To be successful, active proteins must be directly attached to the optical fiber surface.

**Figure 1.**
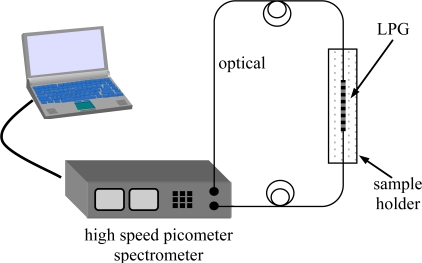
Experimental setup for testing LPFG biosensors for *F. tularensis*. The transmission spectrum was monitored by a high speed picometer spectrometer (Micron Optics SI720; measurement range 1,520–1,570 nm; wavelength resolution 0.25 pm; wavelength accuracy ±1 pm) and recorded by a PC.

Antibody thin films were first fabricated on planar substrates using the LbL/ESA process, and the refractive index change that occurred upon antigen binding was evaluated using spectroscopic ellipsometry. Index changes on the order of 0.02 were observed, which corresponded to typical LPFG wavelength shifts of 20–30 nm. The LPFG surface was prepared as above and a polyelectrolyte film assembled on the surface of a LPFG using PAH, PDDA, and PSS, prior to protein assembly. Because the polymer chain was flexible, it was free to orient its geometry with respect to the substrate, so a relatively low-energy, stable configuration was achieved. By varying the thickness and refractive index of this layer, sensitivity could be maximized by controlling the penetration depth of the optical fiber evanescent wave. Following the coating of the LPFG with the polymer precursor film, IgG was attached to the LPFG by self-assembly after dilution to 50 μg/mL in 0.05 M HEPES buffer. The sensor was then exposed to dilutions of a stock suspension of irradiated cells of *F. tularensis* (10^7^ CFU/mL), and the change in peak wavelength was measured. Sensor output was monitored during film fabrication and antigen binding using the experimental setup shown in Figure 1.

### 2.4. Multi-Cavity Fabry-Pérot Interferometric Sensor Fabrication and Operation

**Figure 2.**
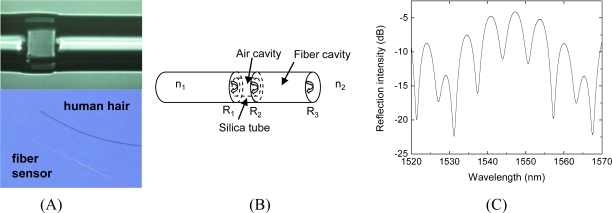
Multi-cavity FP sensor structure and representative interference spectrum.

A Fabry-Pérot interferometer is formed by the reflection of two or more coherent beams from two points separated in space. In an extrinsic fiber Fabry-Pérot interferometer (FFPI), these reflections are induced by the change in refractive index at the interface between the fiber and the surrounding material, and the entire interferometer is contained within the tip of a standard optical fiber (125 μm in diameter). A conventional FFPI consists of an input fiber, an air cavity subject to perturbation by the substance of interest, and a second reflecting surface, which may be either a second fiber or a diaphragm [15–17,19,22], forming a two-beam interferometer. A single cavity FFPI may also be formed by generating an air-filled microgap between two spliced optical fibers [37]. Such sensors are intended to observe the perturbation of only a single cavity. Therefore, the reflections from the interfaces other than the two endfaces of the one cavity are intentionally minimized to reduce the multiple interferences. In contrast, the multi-cavity FFPI sensor contained three reflecting surfaces (R_1_, R_2_, and R_3_ shown in Figure 2(B)) by utilizing the reflection from the outer surface of the reflection fiber (R_3_). The fiber exhibited an interference spectrum more complex than the simple sinusoid of two-beam interferometers, providing enhanced resolution and the ability to detect events within nanoscale thin-film cavities on the endface, as well as the air cavity. Each cavity length can be determined by demodulation of the spectrum. The overall fringe pattern of the sensor is due to the interference between the reflection signals of the three fiber-air interfaces; the lower frequency envelope shown in Figure 2(C) is due to the air cavity. The peak/valley tracing method may not be directly applied to the multicavity sensor because of the multiple interferences involved. Both of the cavity lengths can be demodulated with 0.1 nm resolution using a matrix optics model.

When a thin film is assembled on the fiber endface at R_3_, it can be estimated as an extension of the fiber cavity when the refractive index of the film is similar to the fiber. The biosensing principle is based on the measurement of optical thickness changes of this fiber-film cavity caused by antigen binding/DNA hybridization. Since the optical thickness of the fiber cavity is also dependent on temperature due to the thermo-optic effect and thermal expansion, a key advantage of the multicavity structure is the integrated compensation provided by extracting the temperature information directly from the multicavity structure [38]. Thermocouples as separate devices can also be used in temperature compensation, but the built-in temperature compensation from the multicavity structure results in simple but robust structure and miniature size.

Multi-cavity FFPI sensors (Figure 2) were fabricated by first splicing 50 μm ID/126 μm OD silica capillary tubing to a single-mode fiber (Corning SMF-28). By cleaving the tubing and splicing it to another single-mode fiber, a Fabry-Perot cavity was formed by the reflections from the two fiber-air interfaces. Another cavity was then created by cleaving the second fiber, as shown in Figure 2(B). In designing the fiber and air cavity lengths, two trade-offs were considered: reducing the fiber cavity length to reduce the thermo-optic effect or increasing fiber cavity length for better visibility of the interference spectrum; increasing air cavity length for better temperature compensation or decreasing air cavity length to reduce coupling loss. Air cavities of greater than 20 μm are required to eliminate misalignment of the cavity endfaces; lengths of 30–40 μm were selected to provide both good temperature sensitivity and low coupling loss. The fiber cavity length must be greater than the air cavity length by enough distance to allow sufficient frequency separation of the coupled air cavity, fiber cavity, and air/fiber combination signals, similar to the demodulation of a frequency-based multiplexed sensor, as described elsewhere [38,39]. Cavities in the range of 100–150 μm are sufficient to meet this requirement. The air and fiber cavities were fabricated using conventional fiber cleaving and splicing tools (Sumitomo, Type-36). Air cavity length is limited by the cleaving step to 10 μm or greater and is highly repeatable when performed under a microscope; the fiber cavity requires the same process and thus the same limit. The sensing surface at R_3_ was then modified using the LbL/ESA process described above.

**Figure 3.**
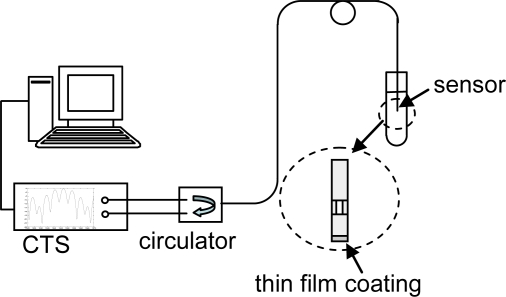
Schematic of multicavity FP sensor system for biosensing applications.

The sensor was inserted into the sample under test for a specified length of time (5 min for DNA tests), removed and rinsed, and the output reflection spectrum was recorded. The reflection spectrum of the sensor was monitored by a component testing system (CTS, Micron Optics SI720), which utilizes a low-noise fiber ring laser as the light source (Figure 3). The analyzer offered a wavelength measurement in the range of 1,520–1,570 nm with 1 pm accuracy (Figure 2(C)). A fiber circulator (AC Photonics) was connected to separate transmission and reflection signals. As DNA hybridization or antigen binding occurred, the reflection spectrum was shifted. By fitting the spectrum to the appropriate mathematical model, the optical thickness change in the sensing surface was obtained, which was dependent on the test sample concentration [38,40]. As the refractive index of the sensing surface is similar to that of the fiber cavity to which it is attached, optically they can be treated as a single cavity. While this system is suitable for laboratory testing, to improve portability and reduce system cost the multicavity sensor may be incorporated into the white light interferometric fiber optic sensor systems developed in our lab [41]. An LED as the low-cost broadband light source and a fiber spectrometer as the detector can provide high stability, high sensitivity, low cost, and compact size.

### 2.5. DNA Immobilization and Hybridization

Detection and differentiation of *F. tularensis* subsp. *tularensis* (strain TI0902) and subsp. *holarctica* (strain LVS) was accomplished using a single-mode multi-cavity FFPI sensor. Sensors were prepared by depositing seven polymer bilayers onto the fiber tip followed by attaching one of two DNA probes: (a) a 101-bp probe from the *yhhW* gene (locus tag FTT_1266c) unique to type A strains (schu4-330 GenBank accession NC_006570), or (b) a 117-bp probe of the *lpnA* gene (locus_tag FTT_0901), common to both type A and type B strains. DNA solutions were boiled for 5 min and immersed in 70% ethanol at 0 °C prior to assembly. The oligonucleotide probes were immobilized onto the surface of the fiber tip by LbL/ESA, using procedures outlined above. Hybridization experiments were performed at room temperature by immersing the optical fiber sensor in homogeneous test solutions. Cavity length analysis was performed both *in situ*, and following a rinse step to remove unhybridized DNA. Each data point was calculated from an average of 300 OPD measurements.

Homogeneous solutions of the 26-base oligonucleotide probes shown in Table 1 (128–152 μM, pH 5.5, 0.02 M NaCl) were used to evaluate the specificity of the FFPI sensors. When bacteria were used as a source of DNA, 2 × 10^8^ cells/mL were lysed in boiling water for 5 min, insoluble material sedimented by centrifugation, and dilutions of the supernatant used in assays. The control DNA sequence consisted of a random base-pair sequence.

### 2.6. Sensitivity Measurement of the FFPI Assay

To evaluate the sensitivity of the multi-cavity FFPI sensors for small quantities of target DNA, sensors were prepared as above and hybridization observed in successively greater dilutions. The solutions of ssDNA-B, ssDNA-C, ssDNA-D, and ssDNA-E (0.02 M NaCl, pH 5.5) were used at concentrations 53 to 152 μM.

## 3. Results and Discussion

### 3.1. LPFG Immunosensor Detection of *F. tularensis*

**Figure 4.**
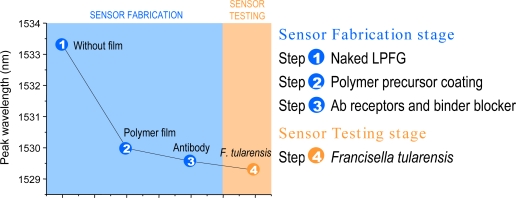
LPFG output wavelength change during film fabrication and sensing validation, indicating the peak wavelength (or output signal) at each step during sensor testing: Steps (1) Bare LPFG (no coating was deposited); (2) LPFG coated with polymer precursor film; (3) Complete sensor with immobilized receptor IgG; and (4) Sensing output indicating the presence of *F. tularensis.*

In order to demonstrate the capability to immobilize proteins directly on the fiber surface that are capable of binding effectively with *F. tularensis* cells, an optical fiber biosensor was developed using a standard transmission mode LPFG of length 15 mm and period 260 μm and incorporating the IgG fraction of antiserum to *F. tularensis*. Sensor output during film fabrication and testing of a solution containing *F. tularensis* cells was monitored (Figure 4). The capability to deposit active IgG directly onto the optical fiber surface in a nanostructured film with controlled optical properties was clearly demonstrated, and the presence of at least 10^5^ CFU of *F. tularensis* cells was readily detected from the decrease of peak wavelength caused by binding of specific antigen. When the immobilized sensor coated with antibody alone was exposed to only saline, no change in output wavelength was observed (not shown). These results were obtained with a standard transmission mode LPFG configuration. The interference fringes produced by a pair of identical LPFGS offer much greater sensitivity than a single LPFG [42]. The coupled cladding mode is mostly guided through the cladding and then re-coupled to core mode by the second LPFG, while the uncoupled core mode power continues to propagate through the core region. These two optical paths effectively create an in-fiber Mach-Zehnder interferometer. To improve sensitivity in future tests, this scheme will be implemented as a reflection mode interferometer [43] in which the light passes through the same LPFG in opposite directions, resulting in a similar effect as two identical LPFGs.

Temperature sensitivity of such LPFG sensors is a concern for refractometry-based applications. Careful photosensitive fiber selection or direct adjustment of fiber dopants can reduce the fiber sensitivity, or it can be compensated by incorporating a temperature-sensing intrinsic Fabry-Perot interferometer (IFPI) into the optical fiber probe [44].

### 3.2. Oligonucleotide Immobilization on the FFPI Endface

**Figure 5.**
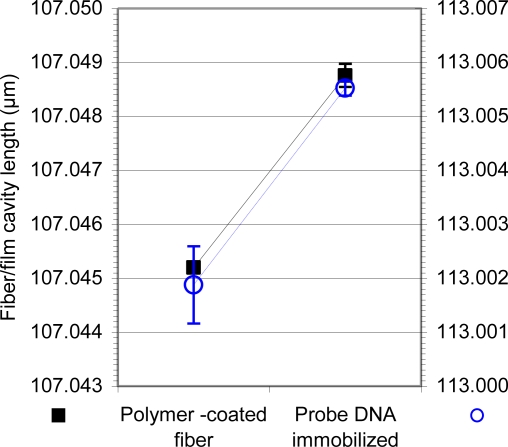
Immobilization of a representative ssDNA probe on FFPI. The ssDNA-A probe was used at 154.17 μg/mL and immobilized at pH 5.5. The immersion time was 4 min. The signal processing instrumentation was capable of detecting cavity thickness changes of 0.1 nm, indicated by the minor tick marks on the y-axes. The assay was performed twice in triplicate. ▪, polymer-coated fiber; 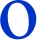, DNA-coated fiber

The FFPI was developed as described in the Experimental Section. Because of its sugar phosphate backbone, DNA is considered an anionic polyelectrolyte that can be immobilized onto the surface of the 125-μm diameter fiber tip by LbL/ESA. The 26-base ssDNA-A oligonucleotide probe to *F. tularensis* (Table 1) was immobilized on the fiber tip by electrostatic self-assembly [24,29,37]. Representative cavity thickness changes resulting from immobilization of the ssDNA-A probe are shown in Figure 5. In the case of either a polymer-coated fiber or DNA coating, self-assembly occurred over a 4-min immersion time.

The resulting cavity thickness changes (Figure 5) demonstrated stable and consistent immobilization of the probe on the FFPI. This was the final step in sensor preparation. When the sensor was employed in target detection, an additional increase in cavity length was expected to occur on hybridization or antigen binding, whereas no increase would indicate the target was not present. The signal processing instrumentation was found capable of detecting cavity thickness changes of 0.1 nm (Figure 5).

### 3.3. Sensitivity of the FFPI Sensors

**Figure 6.**
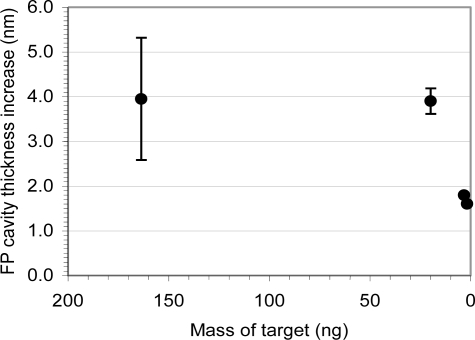
Hybridization of small quantities (1.7–163.8 ng) of ssDNA-B sequence to the ssDNA-A probe. The signal-processing instrumentation was capable of discriminating cavity thickness differences of 0.1 nm, indicated by the minor tick marks on the y-axes. The detection limit is currently on the order of 1 ng.

Small quantities (1.7–163.8 ng) of target DNA (ssDNA-B) hybridizing to the ssDNA-A probe were used to study the sensitivity of the assay. Representative hybridization-induced fiber cavity thickness changes, and the dependence of the optical cavity changes on the mass of the target sequence in the sample are shown in Figure 6. These results demonstrate that specific detection of ng quantities of target DNA sequence are possible following short (∼5 min) hybridization times, without PCR amplification.

### 3.4. Specificity of the FFPI Sensors

**Figure 7.**
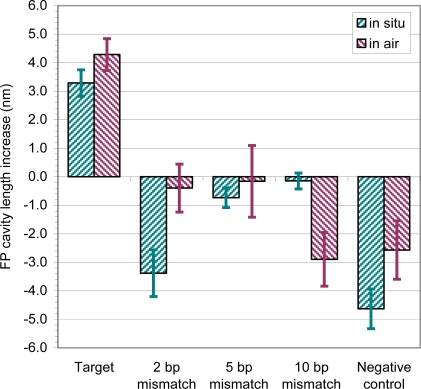
Detection of hybridization of 26-mer target to immobilized probe. Probe: 26-mer ssDNA-A immobilized on FFPI sensor tip; target: ssDNA-B, 53 μM; 2 bp mismatch: ssDNA-C, 138 μM; 5 bp mismatch: ssDNA-D, 152 μM; 10 bp mismatch: ssDNA-E, 145 μM; and negative control: 22-mer random ssDNA-F, 128 μM. A positive slope between probe immobilization and target detection indicates a positive result.

The 26-base ssDNA-A oligonucleotide probe (Table 1) was immobilized on the fiber tip as described above. Specificity of the assay was tested using the five target DNA sequences shown in Table 1 (ssDNA-B, ssDNA-C, ssDNA-D, ssDNA-E and ssDNA-F). The immobilized ssDNA-A probe was hybridized with 53 μM of target DNA (ssDNA-B), which was 100% complimentary to the probe. This resulted in a positive optical thickness increase of 4.2 nm (Figure 7). In contrast, hybridization with target sequences ssDNA-C (containing a 2 bp mismatch), ssDNA-D (containing a 5 bp mismatch), ssDNA-E (containing a 10 bp mismatch), and ssDNA-F (negative control of random DNA sequence) at concentrations of 128–152 μM failed to produce a positive thickness change (Figure 7).

### 3.5. *F. tularensis* Subspecies Differentiation Using FFPI

**Figure 8.**
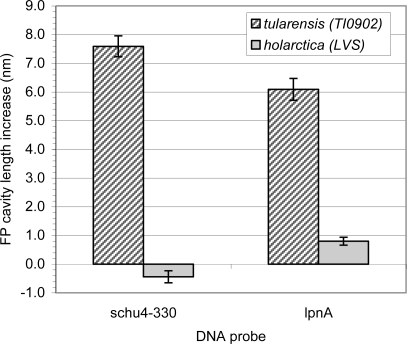
Differentiation of *F. tularensis* strains TI0902 (type A) and LVS (type B) using FFPI sensors. The probe schu4-330 was unique to type A strain TI0902, whereas, the probe lpnA was common to strains TI0902 and type B strain LVS. The sensors measured a change in optical cavity length that correlated to a given concentration of the target. Although the sensitivity of probe lpnA for strain TI0902 was greater than for LVS, the positive slope for both strains indicated both strains were reactive.

Detection and differentiation of *F. tularensis* subsp. *tularensis* (TI0902) and subsp. *holarctica* (LVS) were demonstrated using a single-mode multi-cavity FFPI sensor. The oligonucleotide probes schu4-330 and lpnA, which are unique to type-A strains and common to both type A and B strains, respectively, were individually immobilized on the tip of a FFPI. When the tip immobilized with schu4-330 was hybridized with irradiated cells, only the probe incubated with type A strain TI0902 displayed an increase in cavity thickness. This observation suggested that probe schu4-330 could be used to distinguish type A strains from type B strains of *F. tularensis.* When the tip immobilized with probe lpnA was incubated with irradiated cells, both strains displayed an increase in cavity thickness. This observation confirmed the successful use of this approach in detecting *F. tularensis* and in distinguishing *F. tularensis* type A from type B strains (Figure 8).

## 4. Conclusions

The portable sensing probes described here are well suited for field applications where compact equipment that combines a spectrophotometer and computer is required. Each probe may consist of a number of fibers multiplexed together, each targeting a different DNA sequence or antigen. During specimen testing, antigen binding/hybridization will alter the optical properties of the attached thin film, which will immediately modify the reflection characteristics of the fiber and produce an observable output indicating the presence and concentration of each target antigen. Specificity is provided by the careful selection and optimal immobilization of DNA probes and antibodies; sensitivity is obtained by tailoring the optical fiber and thin-film fabrication process and refining the signal processing algorithm. Sequence-specific DNA detection has become increasingly important as more genomes of high-risk pathogens are sequenced. Significant research has now been devoted to the development of DNA sensors that are sensitive, selective, and relatively simple to use. Typically, optical DNA sensors rely on the attachment of a fluorescent label [45–49]. Even when applied in a fiber optic configuration, most sensors require an external illuminator to excite the fluorescence collected by the optical fiber, limiting this scheme’s application by the need for bulky and expensive instrumentation. The attachment of the necessary labels not only makes the testing time consuming and requires highly trained personnel, but also increases the risk of contamination, human error, or mechanical damage to the probe. In contrast, the assay described here does not require labeling, pre- or post-treatment or amplification of the DNA or antibodies, uses less probe material, and is fast, reusable, and relatively inexpensive. Hybridization of target DNA with complementary capture DNA, or antibody-antigen binding, produces an increase in the fiber cavity length, observable to 0.1 nm with current instrumentation. The use of oligonucleotide probes in these biosensors for detection of *F. tularensis* produced consistent, specific results, without PCR amplification.

## Figures and Tables

**Table 1.** Sequences of *F. tularensis* oligonucleotide probes* used for hybridization experiments.

**Name**	**Purpose**	**Sequence**
ssDNA-A	(probe)	5′-TCCAGACATGATAAGATACATTGATG-3′
ssDNA-B	(target)	5′-CATCAATGTATCTTATCATGTCTGGA-3′
ssDNA-C	(2 bp mismatch)	5′-CTTCAATCTATCTTATCATGTCTGGA-3′
ssDNA-D	(5 bp mismatch)	5′-CTTCAATCTATCTTTTCATGTCTCCA-3′
ssDNA-E	(10 bp mismatch)	5′-CTAGTATGAATGTAATGATGTCTCCA-3′
ssDNA-F	(negative control)	5′-CTCACGTTAATTTTGGTC-3′

* All purchased from Genosys (The control ssDNA-F sequence was randomly designed).
